# Parental mental illness and their offspring’s mental health in Rwanda: neuropsychiatric hospital of Rwanda

**DOI:** 10.1186/s40359-021-00633-3

**Published:** 2021-09-04

**Authors:** Donat Rusengamihigo, Jean Mutabaruka, Emmanuel Biracyaza, Olga Magalakaki, Mayssa El’Husseini

**Affiliations:** 1 Department of Clinical Psychology, School of Medicine and Pharmacy, College of Medicine and Health Sciences, University of Rwanda, Kigali, Rwanda; 2 Pogramme of Sociotherapy, Prison Fellowship Rwanda (PFR), Member of Prison Fellowship International, Kigali, Rwanda; 3 Department of Clinical Psychology, Université de Picardie Jules Verne (UPJV), Amiens, France

**Keywords:** Posttraumatic disorder, Depression, Parental Mental illness, Offspring’ Mental Health

## Abstract

**Background:**

Offspring of the parents with mental disorders are at higher risk to have the mental diseases throughout the world. This study examined the association between psychopathology of parents and the mental health of their offspring in Neuropsychiatric Hospital of Rwanda, Butare Branch.

**Methods:**

A cross-sectional study made up of case and control offspring was conducted on the case group made up of 80 offspring born to parents with mental diseases and a control group of 80 offspring from parents without mental disease. Hamilton Rating Scale for Depression (HRSD, α = 0.82), Posttraumatic stress disorders scale (PTSD, α = 0.73) and the Test of Psychological Problems (TPP, α = 0.93) were used. STATISTICA version 8 was used for statistical analysis.

**Results:**

Results indicated a significance difference between the two groups on depressive symptoms, psychological problems and PTSD symptomatology. The case group seemed to experience high level symptoms than the control group. Results indicated that, among the offspring born to parents with mental disease, there was a significant correlation between anxiety and depression symptoms (r = 0.71, *p* < .001), PTSD and eating disorder (r = 0.75, *p* < .001), domestic violence and PTSD (r = 0.78, *p* < .001), aggressive behavior and PTSD (r = 0.79, *p* < .001), somatoform disorders and PTSD (r = 0.98, *p* < .001). No significant association between the low self-esteem, depression, anxiety, mental disorders induced drug abuse and PTSD was found.

**Conclusion:**

Offspring of the parents with mental disorders had higher risk to develop mental diseases than the offspring born to the parents without mental diseases. Taking into account the assessment of parents’ mental illness when taking care of the offspring’s psychological disorders is needed in the neuropsychiatric hospital.

## Background

Around one in five minor Offspring has the parent who lives with mental diseases worldwide [1, 2]. Offspring whose parents experience mental disorders have higher risk of psychological and developmental disorders compared to the Offspring whose parent do not have mental disorders [3, 4]. In clinical child and adolescent samples, previous studies have estimated that up to half of the Offspring who undergo treatment for psychiatric illnesses have parents who have been diagnosed with a mental disorder [5]. This development of the mental disorders from the parents to their offspring is one of the factor that makes the mental diseases remain the global burden [6]. It was previously found that the Offspring of depressed parents are more likely at greater risk to develop psychopathology and other disabilities [7]. Other studies stated that the population attributable risk proportions for parents mental disorders was higher in high and upper middle than lower middle income countries and higher for abnormal behaviours than the other disorders [8].

Mental Health has been a challenging problem among Rwandan population especially after 1994 genocide against Tutsi [8]. After 1994 genocide perpetrated against Tutsi au Rwanda; it was noticed that genocide caused the increasing of mental disorders such as depressive disorders, anxiety diseases, psychosomatic disorders, disorders induced to drug use, psychotic disorders and schizophrenia and post-traumatic stress disorders (PTSD) related to a traumatic event and it affects many people [8–10]. Mental disorders affect not only parents but also their offspring [11]. The scholars have recently documented that the rate of mental disorders are elevated in Rwanda with a high prevalence of PTSD ranging from 24.8 to 46.4%, depressive disorders ranging from 15.5 to 46.4%, and anxiety disorders at 58.9% [12]. For the neighbour countries to Rwanda, the incidence of mental disorders is highly prevalence. In Uganda the prevalence of various mental disorders is high with 9.3% for depression, 8.5% for anxiety, 4.9% for bipolar disorders, and 1.5% for schizophrenia [13, 14]. In Tanzania, the common mental disorders (CMDs) were low compared to other countries of the same region [15].

In fact, mental disorders within parents is one of the negative emotional experiences accompanied by several predictable physiological, social, cognitive, emotional and behavioral changes on their offspring living with them [11, 12]. Most recent studies conducted on the intergenerational transmission of psychopathology indicated that the mothers might transmit psychopathologies to their offspring. They found that the Offspring of the mothers with psychopathology had risk to develop psychopathology. The same studies indicated that depressed mothers differ from non-diseased mothers in that former are more negative, less positive, less contingently responsive, and more disengaged in integration with their siblings [13].

Moreover, mental disorders refer to a mental or behavioral pattern that cause either suffering or a poor ability to function in ordinary life [14]. A mental illness can be defined as a health condition that changes a person's thinking, feelings, or behaviors (or all three) and that causes the person distress and difficulty in functioning [15]. Such features may be persistent, relapsing and remitting, or occur as a single episode among the Offspring whose parents have mental psychopathology. The transmission of signs and symptoms from parents to their off-springs vary widely vary and they refer to a wide range of mental health conditions-disorders that affect mood, thinking and behaviors of offspring. The mental psychopathologies includes depression, anxiety disorders, schizophrenia, eating disorders and addictive behaviors [20–22]. Other researchers confirmed the impact of parental mental diseases on their offspring particularly postnatal depression. And they have recently found that parental mental diseases lead to insecure attachment for offspring and this affects cognitive, emotional, social and behavior development of child. Indeed, the offspring with the parents diagnosed mental diseases may develop psychiatric disorders in childhood, adolescence and later adult life [22, 23].

Preceding studies found that the genetic influences occur to the offspring whose parents have been diagnosed mental diseases. The scientific evidence from macro-perspective agree that parental psychopathology in perinatal may lead to child functioning beyond young age. This psychological impact may take a long time from childhood to adulthood [25]. The scholars revealed that offspring from parents with depressive disorders have a greater risk to develop behavioral problems [26, 27]. The evidence state that 1 in 5 offspring live in families with a parent who has mental diseases [28]. Etiologically, not only genetic factors may increase the risk but also the environmental factors or various combination of genetic predisposition and adverse environmental determinants may increase the risk to develop any of mental disorders among the offspring whose one or both parents have mental disorder. Some of the environmental risk factors are perinatal, maternal, family, parenting, socio-economic and personal risk factors [28, 29].

Many people have mental health concerns from time to time. But a mental health concern becomes a mental illness when ongoing signs and symptoms cause frequent stress and affect your ability to function [17]. Recently, the research has stated that the offspring whose parents have mental diseases have a greater risk to develop psychiatric and behavioural problems that may affect the adulthood of the child if no early intervention provided to both parents and psychological supports to the offspring. Preceding studies conveyed that offspring whose parents had psychiatric disorders have a greater likelihood to experience psychological problems, substance use that exacerbate their mental functioning, anxiety and bipolar disorders when compared to those whose parents had not psychiatric disorders [26–28]. Other earlier studies stated that most however these offspring develop different psychiatric pathologies and behavioral problems, psychotherapies combined with medication are provided to them for diagnosing and treating those disorders and then their psychological wellbeing is promoted. In addition to that, earlier studies scrutinized that families whose offspring have those psychiatric disorders are often affected by parental illnesses and the psychiatric disorders of their offspring. They also coincided that these offspring from these families handicapped also become more vulnerable in their respective communities than the Offspring whose families are not negatively affected. Similar studies conveyed that these families that face different vulnerabilities due to psychiatric disorders had a greater odds to experience health problems that include social isolation, financial adversities, and marital cacophony [12, 27]. Previous studies evidently demonstrated that offspring whose parents had mental illnesses have a higher risk to develop psychiatric disorders and behavioral problems that might be confounded with the different factors such as environmental, parent–child adjustment mostly associated with the stages of development of the child, type of mental illnesses of the parent, family awareness of the mental illness, types of therapies provided to the parents, and capacities of adults from the families to provide care and other health supports [12, 35].

Moreover, preceding studies stated that the offspring who parents have psychiatric disorders may develop mental illnesses and behaviors problems that may occur in childhood. These effects might weaken their psychosocial, cognitive, and personality development. They also documented that they might develop health problems such as mental retardation, lower performance in their daily activities and at school, and difficulties in their social relationships [12, 16, 20, 36, 37]. Although the psychiatric disorders and behavioral problems might occur since childhood, they also may occur even in the adolescenthood and adulthood [18, 22]. Earlier studies also presented that not all parents with mental illnesses may develop similar degree of parenting difficulties, but those with chronic disturbance are less likely to be sensitive and competent parenting behaving when compared to those parents without chronicity [24, 39]. It was established that a specific diagnosis is neither an independent nor useful predictor for parenting problems or strengths [12, 24].

Although mental diseases are highly prevalent in Rwanda, the accessibility to mental health interventions remain at low level [25]. To our knowledge and based on the above-mentioned scientific evidence, there is very little knowledge known in the neuropsychiatric hospitals about the association between parental mental health and offspring mental health. Therefore, this study investigated the relationship between parental mental illness and their offspring mental health. The researchers hypothesized that the offspring born to the parents with mental mental disorders have a grater risk to develop mental disorders compared to the offspring whose parents with no mental disorders.

## Methods

### Study design

A cross-sectional study design based on control offspring and case offspring was carried out at Cartate Aergrorum Served (CARAES) Butare which is the only branch of National Neuropsychiatric hospital in Rwanda. This design was based on descriptive, analytical and comparative designs.

### Study settings

Rwanda is the small country located in central Africa. This county which is one of the Eastern African Community members has faced many histories that increased the mental disorders. Since its foundation through the collaboration between Brothers of Charity from Belgium and the government of Rwanda, the only Neuropsychiatric Hospital in Rwanda (CARAES-Ndera) was built for promoting mental health from 1962 and was officially opened in 1972. The current neuropsychiatric health facility that is run by Brothers of Charity has also the capacity to provide hospitalization for the severe cases of mental diseases. Although headquarter of national Neuropsychiatric hospital has been located in Ndera at Kigali City (Capital City of Rwanda), from 1978 it opened its branch named CARAES-Butare located in Huye district near the University of Rwanda. This hospital was built because in the twentieth century, there were many mentally diseases people in the country and they were not offered psychological therapies. Moreover, CARAES-Ndera is the only referral hospital for neuropsychiatric disorders, with more than 288 beds. It provides both medication and psychotherapies for the patients with mental diseases. CARAES-Ndera, Butare Branch, offers ambulatory, acute and sustained care to psychiatric patients, with a special focus on child psychiatry, neurology and psychiatric patients with HIV/AIDS. It was found in 1977 by the Brothers of charity. The mission of the CARAES-Ndera including Butare Branch is to be the regional trusted destination provider of high quality and compassionate health care services accessible to all and to provide comprehensive quality health care to all referral patients and witnessing to the Christ love towards sick person “Deus Caritas Est” [26]. In addition to CARAES-Butare, the control group consisted of offspring from the Village surrounding CARAES-Butare which is Sovu village. Sovu Village is located in Huye district, Ngoma sector, Matyazo Cell. To identify the participants from this community, we defined well the households and participants. The principal investigator has lived there for a long time and knew the participants.

### Study participants and procedures

This cross-sectional study recruited offspring whose parents were seeking psychotherapy and medication at psychiatric setting and the others from the parents with no psychiatric background. These offspring of the second group were recruited from the community surrounding the psychiatric setting in which this research targeted. Before recruiting the offspring, the parents were firstly recruited. The recruitment of the participants was applied between October 2015 and February 2016 from the psychiatric settings and diverse communities located around the target psychiatric setting. About 80 offspring from 80 parents with mental disorders were conveniently recruited from 80 distinct families. The 80 offspring from the parents with no mental disorder were recruited throughout introduction of the local leaders and community health workers (CHWs). All participants were enrolled through a convenience sampling.

### Sample

The sample size of this study was 160 offspring including 80 offspring from the parents with mental disorders (case group) from CARAES-Ndera, Butare Branch and 80 offspring from the parents without mental disorders for comparable group, were recruited from Sovu village which is the community surrounding the CARAES-Ndera, Butare Branch.

### Procedures

A case group made up of eighty (80) offspring whose at least one of the parents was mentally ill including 31 males and 49 females (aged 12 to 40 years, mean = 22 ± 6.4 years) and a control group made up of eighty (80) offspring including 31 males and 49 females (aged between 12 and 40, mean = 24 ± 5.8) participated in this cross-sectional study. Offspring were enrolled regardless whether or not they had psychopathology. In the case group or case parents, we enrolled offspring of parents with psychotic disorders, mood disorders, bipolar disorders, and anxiety disorders. Offspring from the control groups or control parents, we recruited offspring whose parent had not psychiatric disorder. Offspring of parents with psychiatric disorders were recruited through the contact of their parents with the neuropsychiatric hospital. To ensure that control offspring were matched with offspring of affected parents, we selectively enrolled control offspring from the same community with the offspring whose parent had psychopathology (they were neighbors).

The eligible offspring in the control groups included 31 males and 49 females who were aged 12–40 years and their parents were aged 45 and 65 years (mean 50 ± 4.1 years). In case group, the study included the offspring whose parents were diagnosed PTSD, Major Depressive Disorder (MDD), psychotic disorders especially Schizophrenia, Bipolar disorder, Drug induced disorder, and Somatoform disorders. All their parents seek psychotherapies and medication at CARAES-Ndera for more than 6 months. To interview the offspring whose parents had mental disorder, the patients were asked if they accept that the researcher interview their offspring. After providing the permission, the researcher and offspring’s guardian invited the child to come at the clinic for participating in the research. For control group, the study included the offspring whose parent had not mental disease during the research. To identify these offspring, the research worked with the Community Health Workers (CHWs) and the local leaders from Huye district through the leader in charge of Health Department to map the families that have parents with no mental disease. All families that availed the eligible offspring were selected in the community surrounding the target clinical setting.

All questions were translated into Kinyarwanda (mother tongue of the participants) from English language by two people who write and speak well both Kinyarwanda and English. These translators were also psychologists and not parts of the authors’ team. Back translation process was performed for enhancing the validity of the selected measures and ensure the quality of the translation. Concerning adaptation of research questionnaires for offspring, the materials used in this study were adapted by the research investigators and then validated to assess how reliable and valid they were before using them in the data collection.

We assert that all procedures that contributed to this research comply with the ethical standards of the relevant and institutional committees on human experimentation and with the Helsinki Declaration of 1975, as revised in 2008 [40]. The study was reviewed and approved by the Institutional Review Board, College of Medicine and Health Sciences (IRB/CMHS) at the University of Rwanda and Hospital’s Program review committee. We received the oral and written informed consent forms from the offspring who were aged more than 18 years since they had capacity to provide them. For the offspring who did not have the capacity to make an informed consent form decision because they were aged less than 18 years, their parents or guardians provided written informed consent form and their offspring provided assent to take part in this research. Confidentiality and voluntariness of the participants were ensured. Participants were allowed to withdraw from the study at any time without providing any reason. Whoever developed the emotional problem was provided the psychological supports by the trained psychologist and the mental health providers of the neuropsychiatric hospital.

## Materials

Data collection was conducted by a trained researchers whose educational background was Clinical Psychology. Data were collected by two data collectors under supervision of the main author. Socio-demographic questionnaire and psychometric measurements were used for collecting accurate data.

### Hamilton Depression Rating Scale (HDRS)

Hamilton Rating Scale for Depression abbreviated as HDRS (also known as the HAM-D) is the psychometric tool used for assessing the depressive symptomatology. The original version of HDRS which is the most widely used clinician-administered depression assessment scale contains 17 items (HDRS-17) pertaining to symptoms of depression experienced over the past week [27, 28]. Although the scale was designed for completion after an unstructured clinical interview, there are now semi-structured interview guides available. The HDRS was originally developed for hospital inpatients, thus the emphasis on melancholic and physical symptoms of depression. A later 21-item version (HDRS-21) included 4 items intended to subtype the depression, but which are sometimes, incorrectly, used to rate severity. A limitation of the HDRS is that atypical symptoms of depression such as hypersomnia, hyperphagia are not assessed by this psychometric measurement [41, 42]. For the subjects who score 0 to 7 are considered to be normal whereas who score 20 and more indicate moderately severe depression. This psychological test evaluated depressive symptoms among offspring from healthy families (control group or control offspring) and from offspring whose parents are mentally ill (case group or case offspring). In the current sample, this psychometric was consistent. This was similar to the prior studies that indicated that it is valid and reliable in Rwandan samples of psychiatric patients and non-psychiatric patients [30]. Although the HDRS was designed for the adults, few studies have established that it is also suitable for the adolescents and young adults for detecting the severity of depression [42, 43, 44].

### Post-traumatic Stress Disorder (PTSD) Checklist PCL

Post-traumatic Stress Disorder (PTSD) Checklist (PCL) is one of the most commonly used psychometric measurement for assessing 17 symptomatology of PTSD found in the DSM-V [16, 29]. This measurement has excellent test–retest reliability over a 2–3-day period. Internal consistency is very high for each of the three groups of items corresponding to the DSM-IV symptom clusters as well as for the full 17-item scale. The PCL correlates strongly with other measures of PTSD, such as the Mississippi Scale, the PK scale of the Minnesota Multiphasic Personality Inventory-2 (MMPI-2), and the Impact of Events Scale, and also correlates moderately with level of combat exposure [30]. The scores of this psychometric instrument range between 17 and 85 [43]. This test was used to assess the symptoms of PTSD among offspring from balanced families (Control group) and among offspring whose parents are mentally ill (case group). Besides, the respondents in this self-report are asked to rate the degree to which they were bothered by the symptoms in the past month. The Likert Scale is used from 0 (not at all) to 4 (extremely) [29, 31]. In the previous studies conducted in Rwanda, the PCL was found to be valid and consistent in both psychiatric patients and general population and among genocide survivors. This instrument was firstly validated by the Pham et al. [48] and they designed the first version was translated into Kinyarwanda in which we used. They produced the Kinyarwanda version from English using forward-back translation method and fielded in a large sample of individuals without facing any difficulty. Although they did not present the psychometric properties for this scale, the other researchers in Rwanda indicated that the PCL is valid and reliable with sufficient Alpha of Cronbach of 0.93 [11, 45, 46]. Although the PCL was developed to be used in adults, few studies conveyed that it is suitable to be used among adolescents and young adults [48]. The psychometric validation of the DSM-5 (PCL) was recently done by Sengesho et al. in Rwanda (the relating research article is accepted for publication in the International Journal of Behavioral Sciences [IJBS]) and they found that the PCL might be administered among young people since it has sufficient psychometric properties. In their study, the mean age was 23 (near 22 years) and the internal consistency was satisfactory (α = 0.75) [49].

Socio-demographic and clinical questionnaires were used in this study for describing the participants and demonstrate the psychological problems.

To assess psychological problems, Test of Psychological Problems (TPP) Likert scale was employed. This questionnaire contains forty-four items (44 Items) that were scored using a 5-point scale (0 = never; 1 = rarely, 2 = occasionally, 3 = often, 4 = very often). The items from 1 to 17 were about mood disorders; for example: “*I feel very happy or act silly in a way that’s unusual*”. Items 18 to 30 were about psychotic disorders; example: “*I feel inappropriate laughter or crying*”. Items 31 to 44 were concerned on anxiety disorders and mental health related disorders; example: “*I feel worry about developing serious illness*”. Concerning the validity and reliability of the used psychometric measurements, before collecting data using the current psychometric measurement in the target setting, these scales were validated in the participants whose characteristics were the same to the target population of this study. Thus, the tools were administered among the patients with psychiatric disorders at Kabutare District Hospital near the CARAES-Ndera. In these samples, the consistency for all administered psychometric measures were adequate (Alpha of Cronbach of HDRS-21, α = 0.82, PCL, α = 0.73 and for PPT, α = 0.93).

### Statistical analysis

Descriptive and analytical statistical analyses were conducted using STATISTICA version 8. Electronic Statistics Textbook. In descriptive statistics, the means, median, standard deviation and variances were computed for describing the participants and assessing the level of severity of symptomatology of depression. A hierarchical multiple regression analysis was then conducted to study the relative contribution of each parent mental illness variable to their offspring’s mental health. In the analysis, we included fixed effects of age, and biological sex as covariates. The contribution of each independent variable was reported as the coefficient β, the statistic t-test and the *p* value of 5%. Significant *β* values were retained at α threshold of *p* < 0.05, with an intensity of *p* ≥ 0.2. To compare the two groups of offspring whose parents had mental disorders and the offspring whose parents had no psychiatric disorder, the independent sample t-test was computed with the 95% confidence intervals.

## Results

The results indicated that the mean age of the sample was 17 years (SD = 4.3) with 40 offspring (50%) males and 40 offspring (50%) females. The majority (52.5%) of the research subjects reported did not have an occupation. Majority also attended one year of primary school (18.8%), three (11.3%) and five years (11.3%) of primary schools. Results showed that majority of respondents were aged 12 and 13 years, and 23 years (Table 1).

**Table 1:** Descriptive analysis of the participants

Characteristics	Number	Percentage
Age		
Age (mean, SD, Median, range)	(Mean = 17, SD = 4.3, Median = 16, range 11–26)	
Sex		
Male	40	50
Female	40	50
Occupation		
No job	42	52.5
Student	24	30
Agricultural activities/livestock	11	13.8
Other	3	3.7
Offsprings from the parents with mental disorders		
Yes	40	50
No	40	50
Marital status		
Single	35	43.8
Married	31	38.8
Separated/divorced	14	17.4
Years of education		
Years (Mean, Median, SD)	(Mean = 4.5, Median = 4, SD = 3.1)	
Years of education		
0 year	5	6.3
1 year	15	18.8
2 years	8	10
3 years	9	11.3
4 years	4	5
5 years	9	11.3
6 years	5	6.3
7 years	8	10
8 years	7	8.8
9 years	7	8.8
10 years	1	1.3
11 years	1	1.3
12 years	1	1.3
Age of participants		
12 years	11	13.8
13 years	15	18.8
14 years	7	8.8
15 years	4	5.0
16 years	5	6.3
17 years	6	7.5
18 years	4	5.0
19 years	3	3.8
20 years	4	5.0
21 years	6	7.5
22 years	2	2.5
23 years	6	7.5
24 years	1	1.3
25 years	3	3.8
26 years	3	3.8

SD, standard deviation

The cut-offs were used to determine the significance of psychological problems. The results indicated significant symptoms of mental problems among the case group. HRSD was higher with the mean of 27.4 (SD = 5.3, cut off ≥ 23); for PCL (Mean = 30.1, SD = 8.5, cut-off ≥ 44) and for TPP (mean = 64.5, SD = 8.8, cut-off ≥ 44), but for the control group the results showed TPP (Mean = 58.9, SD = 15, cut off ≥ 23); PCL (Mean = 33.2, SD = 6.2, cut off ≥ 44) and HRSD (mean = 21.4, SD = 6.8, cut-off ≥ 44). These results showed that the case offspring had a higher level of depression, perceived stress than the control offspring **(**Table 2).

**Table 2:** Descriptive analysis of the psychometric instruments

Scale	Study groups	Mean	Standard Deviation
TPP	Case group	64.5	8.8
	Control group	58.9	15
HRSD	Case group	27.4	5.3
	Control group	21.4	6.8
PCL-5	Case group	30.1	8.5
	Control group	24.9	6.2

Case group, offspring who parents had mental disorders; Control group, offspring from the parents with no mental disorder; HRSD, Hamilton Depression Rating Scale; TPP, Test of Psychological Problems

Table 3 illustrates that the mean difference of the case and control groups in all psychometric measures were different. Using the Levene’s test of equality of variances we found that the variances of the used psychometric instruments in control and case groups were significantly different where we greatly found F-test of 4.75 (*p* = 0.032) for PCL, F-test of 9.52 (*p* = 0.003) for TPP and F-test of 4.26 (*p* = 0.042) for HRSD. Results indicated the significant mean and variances differences between the case and control group on depressive symptoms where the mean of depression was higher in the offspring whose parents had mental disorder. For PCL, the offspring who had parents experiencing mental disorders also had a greater level of PTSD while for TPP they also had a greater level of symptomatology. The results indicated that there was a significant difference in means for HRSD (mean-difference = 6, t = 3.21, *p* < 0.01), for TPP (mean-difference = and 5.6, t = 2.04, *p* = 0.044), and for PCL-5 (mean-difference = 5.3, t = 17.8, *p* = 0.002). Therefore, parental mental diseases seemed to affect mental health of their offspring.

**Table 3:** Difference in symptomatology

Scales	Levene's test		T-test for equality of means				
	F	*p* value	t	*p* value	Mean difference	95% CI	
						Lower	Upper
TPP							
Equal variances assumed	9.5	0.003*	2.04	0.044*	5.6	0.14	11.11
Equal variances not assumed				0.045*	5.6	0.12	11.13
HRSD							
Equal variances assumed	4.3	0.042*	4.35	< 0.001***	6	3.21	8.64
Equal variances not assumed				< 0.001***	6	3.21	8.64
PCL							
Equal variances assumed	4.8	0.032*	3.2	0.002**	5.3	1.93	8.57
Equal variances not assumed				0.002**	5.3	1.93	8.57

PTSD, posttraumatic stress disorder; HRSD, Hamilton Depression Rating Scale; TPP, Test of Psychological Problems; PCL, PTSD Checklist; F, F-test; t, t-test

(*) Significance at 0.05; (**) Statistical significance at *p* < 0.01; (***) Indicates highly statistical significance at 0.001

The study revealed that demographic variables such as sex, age, education, professional, and localization were not associated to mental illnesses neither in offspring nor in their parents. The demographic variables didn’t explain the symptoms of depression and PTSD, nor psychological problems among offspring whose parents were mentally ill.

The results indicated that, among the offspring born to parents with mental disease, there was a significant correlation between anxiety and depression symptoms (r = 0.71, *p* < 0.001), PTSD and eating disorder (r = 0.75, *p* < 0.001), domestic violence and PTSD (r = 0.78, *p* < 0.001), aggressive behavior and PTSD (r = 0.79, *p* < 0.001), somatoform disorders and PTSD (r = 0.98, *p* < 0.001). No significant association between the low self-esteem, depression, anxiety, mental disorders induced drug abuse and PTSD was found (Table 4).

**Table 4:** Correlations between symptoms in the offspring

Health problems and mental disorders	Psychometric measures	
	HRSD (depression)	PTSD
Anxiety disorder	r = 0.71, *p* < 0. 01**	r = 0.2, *p* = 0.091
Mental disorders induced drug abuse	r = 0.72, *p* < 0. 01**	r = 0.19, *p* = 0. 187
Schizophrenia	r = 0.99, *p* < 0. 01**	r = 0.31, *p* = 0.006*
Eating disorders	r = 0.26, *p* = 0.019*	r = 0.76, *p* < 0.01**
Somatoform disorder	r = 0.25, *p* = 0.028*	r = 0.99, *p* < 0.01**

PTSD, posttraumatic stress disorder; HRSD, Hamilton Depression Rating Scale

^*^Statistical significance at *p* < 0.05; ** High statistical significance level at *p* < 0.001

## Discussion

The main goal of this study was to identify the relationship between parent’s mental illness and their offspring’s mental health in Rwanda. The research question was: “How does parental mental illness affect offspring and families?” The results of this study revealed that the offspring born to the parents with mental diseases are at risk to develop mental diseases. These results are supported by the previous studies that confirmed the offspring of mentally ill parents have a higher risk of developing mental diseases over the course of their daily lives [4, 17]. Our results revealed a high level of psychopathology among offspring of parents with mental disorders and low level of symptoms in offspring of control parents. The risk to develop depression was found to be higher for the offspring who parent has one or more of the following disorders: bipolar disorder, anxiety disorders, schizophrenia, psychosis induced drug use, and depression. These results are relevant with the previous studies [50].There was a significant increase of psychopathology in the offspring from the parents with depression when compared to the offspring from the parents with no psychiatric symptoms. This might be due to the genetic influence of developing psychopathological symptoms among those descendants from the parents who have been diagnosed mental disorders. Our results are in congruence with preceding studies stating that the environment in which youth grow affects their development and emotional mental health as much as their genetic makeup does [51]. The parents with mental disorder can genetically affect their offspring [33].

Results of our study showed that there is a significant link between parental mental disease and development of anxiety disorders and depression disorders among their descendants. These findings collaborate with the aforementioned results that suggested that the offspring of parents with anxiety disorders are more likely to manifest anxiety disorders. The offspring from the parents with both depression and anxiety are more likely to develop similar disorders [34]. Prior studies indicated that parents with mental illness are significant risk factor for offspring abuse and neglect [11]. In concur with prior studies that have established that offspring of parent with mental disorders have greater risk to develop psychopathology themselves [38], our results showed a significant increase of psychopathology symptoms among offspring of parents with depression and PTSD when compare to those with controls.

In correspondence with preceding studies, our results revealed a relationship between parents' psychotic disorders and offspring' mental Health. It would seem that most types of mental illness are supposed to be influenced by a combination of biological and environmental factors [31, 50]. The parents with mental illness impacted offspring’s mental health. The results from our study revealed that the offspring whose parents experienced mental problem may develop the psychological problems. These results are in congruence with prior studies [36]. In concur with prior studies [50], our results revealed that only offspring from the parents with psychotic disorders were significantly affected by psychopathology. Our results are relatively in the same vein with the previous research that indicated that the parent psychopathology increases the risk for their offspring to develop mental disorders and psychological problems [8, 47]. It was revealed that isolation, suicidal attempts and anger in parents with psychotic disorders were associated not only with offspring symptoms but also with other psychopathology such as the anxiety, specific substance use disorder (SUD) and social withdrawing. These associations remained stable even after adjustment for parental co morbidity [36, 49]. It was found that offspring who have a parent with a mental illness are at significantly greater risk for multiple psychosocial problems [19]. Indeed, the offspring of mentally ill parents have higher rates of psychiatric diagnoses in childhood and more likely to show developmental delays, lower academic competence, and difficulty with social relationships [16, 27, 28, 42]. The results of the current study revealed that the offspring whose parents have mental diseases are more likely to have mental problems in adolescence and adulthood since the recruited participants were adolescents and adults, however; their parents were diagnosed having psychopathology when the eligible participants were aged less than 18 years. These results are in line with the prior studies [18, 22].

### Strengths and limitations

The strengths of this study were the enough sample size of the recruited parents and offspring. The methods used were also the strength for the current study. It is worth noting here that the recruitment procedure and the traits of the participants who agreed to take part was the possible limitation of the study. The other strength of this study was that the researchers used the validated and standardized materials for collecting data from the research subjects in both medical settings and community. However, diverse strengths for the study were found, the current study had a number of limitations. As a case–control study design, the participants in control groups (control parents) and case group were not enough for generalizing. Secondly, the study didn’t conduct a study with a large sample size that includes a larger number of controls and cases.

## Conclusion

In conclusion, our study revealed a link between parent’s mental illness and the offspring mental health especially with psychotic disorders. For offspring whose parents had psychopathology, our findings revealed significant correlations between anxiety symptoms and depression, PTSD symptoms and eating disorder, and PTSD, somatoform disorders and PTSD. Our findings reported that the symptoms are elevated among the young offspring of parents with psychiatric disorders when compared to those whose parents had no psychopathology. The results of this study suggested taking into account this known odds in the practical provision of health care but they need to be evaluated to a larger population. The risk of developing mental disorders for the offspring born to parents with mental diseases should therefore be taken into account in the practical provision of mental health care. Based on the results, not all offspring of parents with mental psychopathologies develop mental disorders.

The further study should be conducted on indicating the contribution of the psychological interventions and treatment to the prevention of mental disorders amongst offspring born to parents with mental diseases in Rwanda. There is a need to conduct a longitudinal study among cases and controls and then the study on the prevalence and the risk factors of developing mental disorders for the offspring whose parents have mental disorders in Rwanda is needed. Finally, further studies will need to determine these influences by including mothers, fathers or co-parents in their future research designs. The research recommend the policy makers and the staff of CARAES-Ndera to increase the multidisciplinary work for making together their efforts to implement early interventions to alleviate the transmission of psychopathology risk from mentally ill parents to their offspring.

## Data Availability

All data generated and analysed in this study are included in this work and they are available from the corresponding author whose name Donat Rusengamihigo. They may be shared when reasonable request is provided to him.

## Abbreviations

AIDS: Acquired Immune Deficiency Syndrome
CARAES: Cartate Aergrorum Served
HIV: Human Immunodeficiency Virus
NPH: Neuro-psychiatric Hospital
PTSD: Posttraumatic Stress Disorders
